# Time-Resolved Spectroscopic Study of *N,N*–Di(4–bromo)nitrenium Ions in Acidic Aqueous Solution

**DOI:** 10.3390/ijms20215512

**Published:** 2019-11-05

**Authors:** Lili Du, Zhiping Yan, Xueqin Bai, Runhui Liang, David Lee Phillips

**Affiliations:** 1 Institute of Life Sciences, Jiangsu University, Zhenjiang 212013, China; justailleen@gmail.com; 2 Department of Chemistry, The University of Hong Kong, Hong Kong, China; mcayzp@gmail.com (Z.Y.); xqbai@hku.hk (X.B.); rhliang5@hku.hk (R.L.)

**Keywords:** nitrenium ion, time resolved spectroscopy, resonance Raman

## Abstract

Nitrenium ions are common reactive intermediates with high activities towards some biological nucleophiles. In this paper, we employed femtosecond transient absorption (fs-TA) and nanosecond transient absorption (ns-TA) as well as nanosecond time-resolved resonance Raman (ns-TR^3^) spectroscopy and density function theory (DFT) calculations to study the spectroscopic properties of the *N*(4,4′–dibromodiphenylamino)–2,4,6–trimethylpyridinium BF_4_^−^ salt (**1**) in an acidic aqueous solution. Efficient cleavage of the N–N bond (4 ps) to form the *N,N*–di(4–bromophenyl)nitrenium ion (**DN**) was also observed in the acidic aqueous solution. As a result, the dication intermediate **4** appears more likely to be produced after abstracting a proton for the nitrenium ion **DN** in the acid solution first, followed by an electron abstraction to form the radical cation intermediate **3**. These new and more extensive time-resolved spectroscopic data will be useful to help to develop an improved understanding of the identity, nature, and properties of nitrenium ions involved in reactions under acidic aqueous conditions.

## 1. Introduction

Nitrenium ions are common reactive intermediates with a formal positive charge on nitrogen, which attracts lots of attention due to their biological activities. It has been demonstrated that arylnitrenium ions have shown mutagenic properties as a result of chemical carcinogenesis of aromatic amines [1,2,3,4,5,6,7,8,9]. Falvey and coworkers [10,11,12,13] reported several studies on the chemical and spectroscopic behavior of the *N,N*–diphenylnitrenium ion formed after photoexcitation of the 1–(*N,N*–dipheylamino)–2,4,6–triphenylpyridinium ion (see Scheme S1 in the supporting information), and this phenylnitrenium ion was predicted to exhibit ground state singlet properties with some overlap between an unoccupied nitrogen based *p*-orbital with occupied π-orbitals on the phenyl groups [11,14]. Additionally, Thomas and coworkers reported that the *N,N*–di(4–bromophenyl)nitrenium ion (**DN**) formed after photoexcitation of the *N*–(4,4′–dibromodiphenylamino)–2,4,6–trimethylpyridinium BF_4_^-^ salt (**1**) showed more stability and a much longer lifetime than the unsubstituted *N,N*–diphenylnitrenium ion, the lifetime of which was 124 µs in neat MeCN solution and 6.57 µs in an aqueous solution [13]. Furthermore, our group has determined that the generation of **DN** both in MeCN and near neutral aqueous solution was around 4 ps [15]. The singlet ground state properties of the **DN** was also demonstrated by the nanosecond time-resolved resonance Raman (ns-TR^3^) spectroscopy data, in which the direct fingerprint information of **DN** was obtained. Similar reaction mechanisms to those found in Falvey and coworkers’ previous study were also concluded in the near-neutral aqueous solution, as found by a vibrational spectroscopic study [15].

**Scheme 1:**
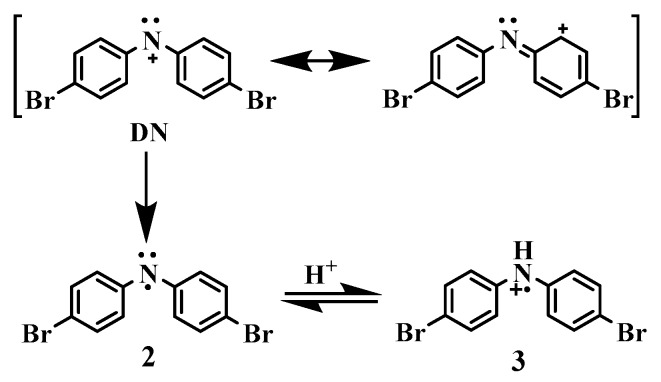
Proposed mechanism of **DN** by Falvey [13].

Unlike the 2-fluorenyl nitrenium ion [9,16,17,18,19,20], the lack of the hydrogen on the positive charge nitrogen on **DN** might show poor acidity for the difficulty in dissociation of a phenyl ring. According to a previous study by Falvey and coworkers [13], the **DN** in an acidic solution should be able to abstract an electron, followed with an abstraction of a proton to form the radical cation (intermediate **3**) as shown in Scheme 1 [15]. However, McClelland and coworkers [21] also mentioned that nitrenium ions were like weak bases in acidic aqueous solution and could accept a proton to form the aniline dication species. Therefore, the reaction mechanisms of **DN** in acidic solutions have remained unclear due to the lack of direct evidence regarding their structure and properties of the relevant intermediates after irradiation.

In this paper, femtosecond transient absorption (fs-TA) and nanosecond transient absorption (ns-TA) were employed to study the spectroscopic properties of **DN** in an acidic mixed aqueous solution. In addition, the structures and characteristics of the relevant intermediates were characterized with ns-TR^3^ spectroscopy experiments, and these results are to our knowledge the first time-resolved vibrational spectroscopic characterization of the diphenylnitrenium ion in acidic aqueous solutions. Furthermore, density function theory (DFT) calculations were also utilized to predict the normal Raman spectra of the possible intermediates, and these results provide new insight into the structure and fingerprint information for those intermediates observed in the acidic aqueous solutions examined in our study.

## 2. Results

### Time-Resolved Spectroscopic Study

The fs-TA spectra of **1** in 1:1 MeCN:1 mM HClO_4_ acidic aqueous solution were acquired and are shown in Figure S1 (left). At early times, from 859 fs to 92.3 ps, an absorption band at 375 nm decreased gradually, accompanied by an apparent peak blue-shifting to 440 from 450 nm. Meanwhile, the feature at 680 nm also intensified during the whole process. The changes during this time period can be attributed to the generation of the **DN** intermediate following the photo-cleavage of the N–N bond, which has been explained in detail in our previous work [15]. In Figure S1 (right), both of the kinetics at 375 and 450 nm were simulated satisfactorily by single-exponential functions (for τ_375_ = 5.9 ± 2.0 ps, τ_450_= 4.0 ± 1.0 ps), indicating that the time constant for the generation of **DN** from the singlet excited state S_1_ of **1** appears to be around 4 ps.

**Figure 1:**
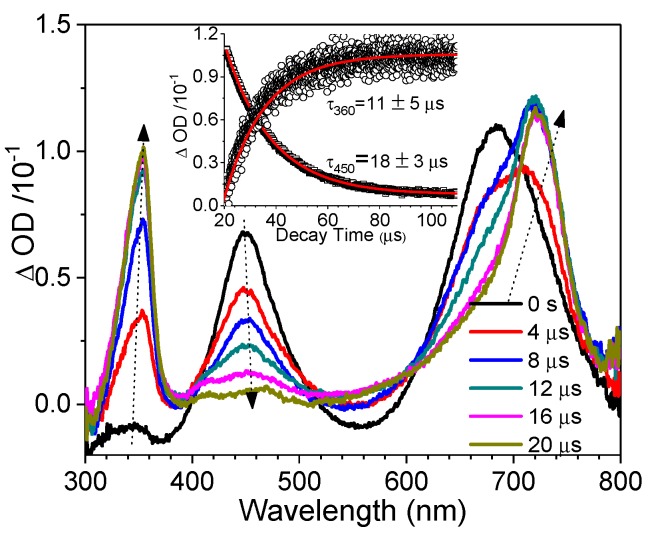
ns-TA of **1** acquired in 1:1 MeCN:1 mM HClO_4_ solution after 266 nm irradiation. The kinetics at 450 and 360 nm are shown in the insert.

In the absence of efficient nucleophilic traps, the nitrenium ion ultimately accepts an electron, which can be suppressed through protonation with a strong acid, producing the radical cation [13]. Similar ns-TA spectra were acquired, shown in Figure 1, which was consistent with results from a previous study by Falvey and coworkers [13]. After excitation, the bands at 450 and 680 nm decreased (decay time is about 18 ± 3 µs) to create the bands at 360 and 720 nm in about 11 ± 5 µs, and this process was assigned to the decay process of **DN** and the generation of radical cation **3**. However, McClelland and coworkers also noted that nitrenium ions can act like weak bases in acidic aqueous solutions and could accept a proton to form the aniline dication species [21]. Therefore, the new species obtained in Figure 1 could be assigned to either a radical cation (**3**) or a dication species (**4**).

**Figure 2:**
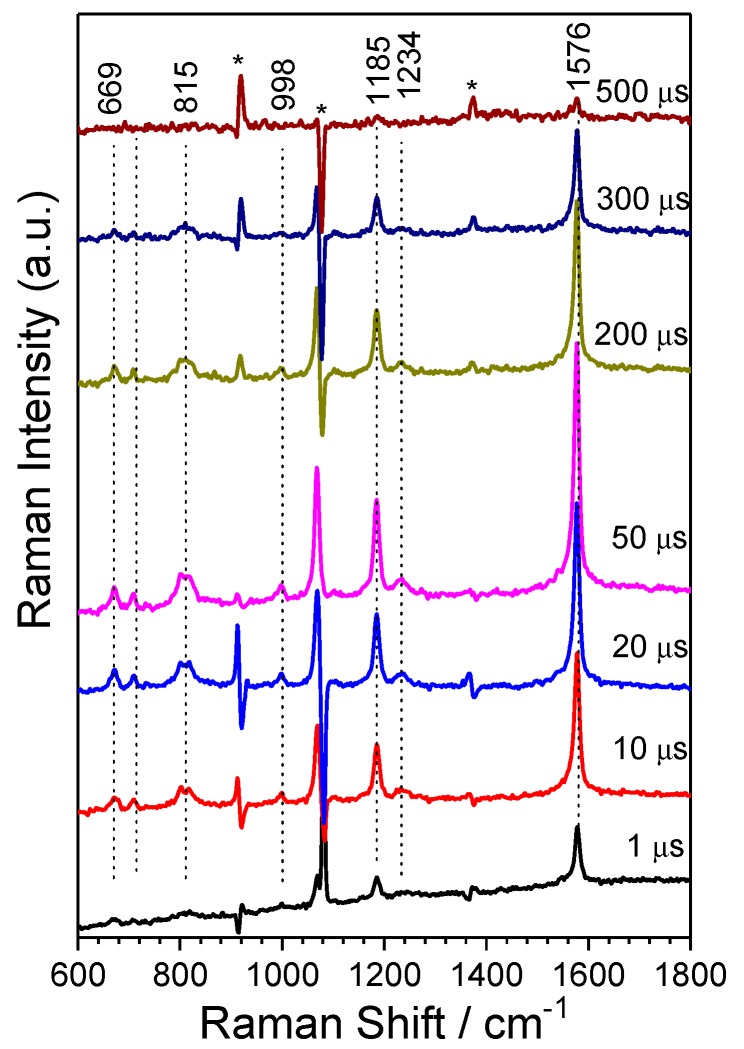
The ns-TR^3^ spectra of **1** produced in 1:1 MeCN:HClO_4_ (1 mM) acquired by pump 266 nm, probe 368.9 nm. The star symbols indicate the regions affected by solvent sub-traction artifacts.

**Figure 3:**
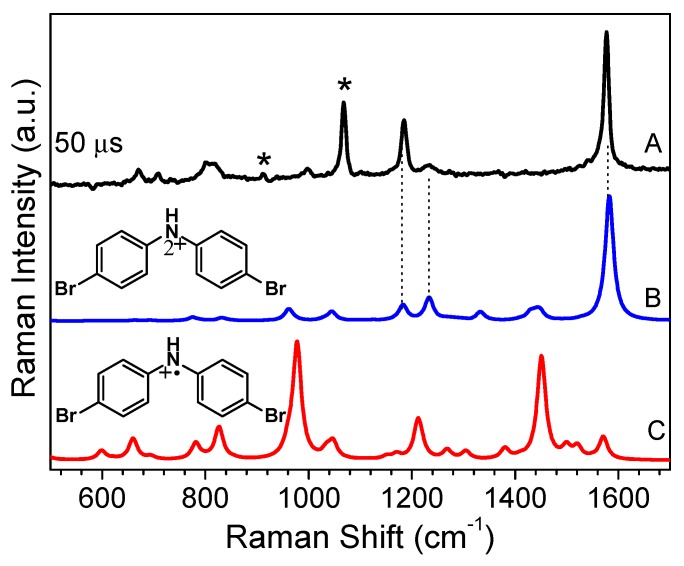
Comparison between the ns-TR^3^ spectra of **1** at 50 µs achieved in 1:1 MeCN:1 mM HClO_4_ solution (pump 266 nm, probe 368.9 nm) (**A**) and the density function theory (DFT)-calculated Raman spectra of intermediate **3** (**C**) and **4** (**B**). The * symbols result from the solvent subtraction artifacts.

**Figure 4:**
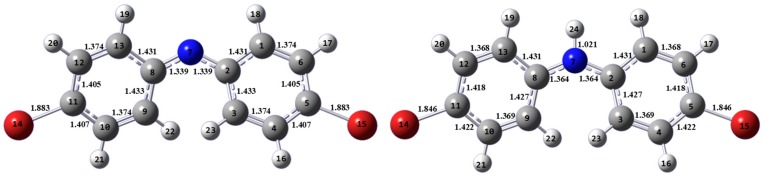
Optimized structures of the N,N–di(4–bromophenyl)nitrenium ion (**DN**) (left) and the dication species **4** (right).

In order to distinguish the vibrational properties of the new species generated for **1** in the acidic mixed aqueous solution after photoexcitation, we used ns-TR^3^ experiments with 266 nm as the pump laser wavelength and 368.9 nm as the probe laser wavelength. Figure 2 displays the ns-TR^3^ spectra of **1** in 1:1 MeCN:1 mM HClO_4_ solution and a new species signal increased after 1 µs and decreased after 50 µs, which is consistent with the observation in the ns-TA results. There are five obvious Raman bands seen in the ns-TR^3^ spectra, which are located at 1576, 1234, 1185, 998, 815, and 669 cm^−1^. As discussed above, both the radical cation intermediate **3** and the dication intermediate **4** are possible intermediates after photoexcitation of **1** in the acidic mixed aqueous solution. Therefore, DFT computations were employed to predict the normal Raman spectra of the radical cation **3** and the dication **4,** and these predicted spectra were compared with the experimental TR^3^ spectrum observed at 50 µs in Figure 3 [22,23,24,25,26,27]. Examination of Figure 3 shows that the calculated Raman spectrum of the dication species **4** (Figure 3B) exhibits significantly better agreement with the experimental TR^3^ spectrum (Figure 3A) in terms of the vibrational frequency patterns of both spectra. All the calculated Raman bands at 1576, 1298, and 1185 cm^−1^ for the dication species **4** are consistent with the experimental results. The characteristic band at 1576 cm^−1^ is mainly due to the intense stretching mode of the phenyl rings and the band at 1234 cm^−1^ can be attributed to the C–N stretching mode. The vibrational band at 998 cm^−1^ can be assigned to the stretching mode of the C–Br. The other three bands at 1185, 815, and 669 cm^−1^ can be attributed to the C–H wagging modes. Compared with our previous study [15], the phenyl ring C–C stretching band of the dication intermediate **4** occurs at 1576 cm^−1^ and it shifts to a higher frequency for the nitrenium ion **DN**, indicating the strengthening of the inter-ring C–C bond of the **DN**. However, the stretching band of C–N occurs at 1371 cm^−1^ for the nitrenium ion **DN** [22] and at 1234 cm^−1^ for the dication intermediate **4**, which is due to the elongation of the C–N bond (from 1.339 to 1.364 Å) of the dication intermediate **4** after protonation as shown in Figure 4, which presents the calculated optimized structure of **DN** (left) and the dication species **4** (right). In Figure 4, we may also notice that the bond length of the C–Br bond shortens from 1.883 Å for **DN** to 1.846 Å for the dication **4**, which results in the upshift of the vibrational band of the C–Br bond to a higher wavenumber, from 985 to 998 cm^−1^ [15]. Thus, the new species observed in the acidic mixed aqueous solution should be the dication intermediate **4** with a lifetime of around 100 µs and this indicates the nitrenium ion **DN** exhibits a basic property in the acidic mixed aqueous solution and has a strong ability for abstracting a proton from the solution to form the dication species first and does not form the radical cation species or the aniline cation species from the **DN** intermediate directly [21]. In the spectra at later delay times, the dication intermediate **4** appears able to accept an electron to produce the radical cation **3** according to a previous study [13]. These new observations and associated information can be utilized to provide deeper insights into the geometries, properties, and chemical reactivity of nitrenium ions.

## 3. Discussion

**Scheme 2:**
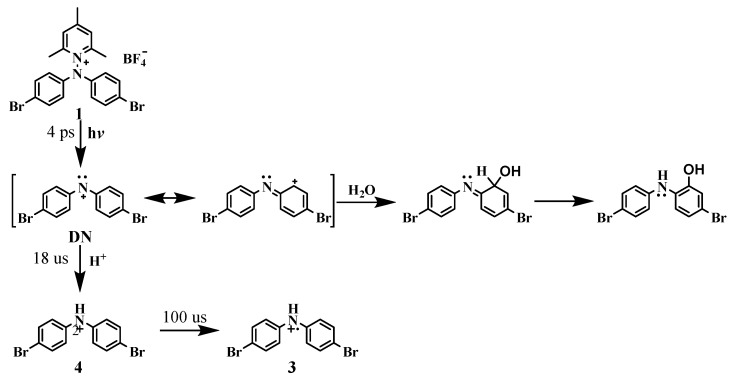
Proposed mechanism for the photochemistry of **1** in different solutions.

The nitrenium ion **DN** was also generated efficiently (4 ps) in the acidic mixed aqueous solution, which was very similar to previous results found in a MeCN solution [13,15]. Unlike the results reported previously [13], the second intermediate involved in the acidic mixed aqueous solution and observed by the ns-TR^3^ experiments should not be the radical cation **3**. As a result, the dication intermediate **4** is more likely to be produced after abstracting a proton for the nitrenium ion **DN** in the acidic mixed aqueous solution, while the radical cation **3** could be formed by accepting an electron after the dication intermediate **4** was formed first. As a summary, the proposed mechanism for the photochemistry of **1** in acidic mixed aqueous solution is shown in Scheme 2. These new and more extensive time-resolved spectroscopic data will be able to help to develop an improved understanding of the identity, nature, and properties of the nitrenium ions involved in the photochemistry of interest. Furthermore, the supplementary results of **DN** in the acidic aqueous solution will provide us with more information regarding the reactivity of the nitrenium ions and be particularly instructive in the study of the reaction towards the biological nucleophiles, such as DNA bases and amino acids under acidic conditions in the future. We note that this is primarily an experimental time-resolved spectroscopic study, and we only calculated the normal Raman vibrational frequencies and did not use the intensity information to distinguish between the different intermediate species observed in the ns-TR^3^ spectra and only utilized the frequency patterns to tell them apart. We further note that this may be an interesting area of study for a theoretical research group in future to examine both the resonance Raman effect and the anharmonic effect on the TR^3^ spectra for these intermediates, so as to gain more insight into their excited state properties associated with the resonance Raman effect and the influence of anharmonicity on the ground state Raman frequencies, but these issues are beyond the scope of the present investigation.

## 4. Materials and Methods

The sample of **1** was synthesized following a literature procedure [13] and spectroscopic grade MeCN (Sigma-Aldrich, Hong Kong), along with 1 mM HClO_4_ (Sigma-Aldrich, Hong Kong) solution, were used for preparing the sample solutions. Unless specified, all the mixed solvent ratios are of volume ratios.

### 4.1. Fs-TA and Ns-TA Experiments

The fs-TA and ns-TA experiments used the same experimental setups and methods previously described in earlier studies [23,24,25]. The fs-TA spectra (Ultrafast System, Sarasota, FL, USA) were collected on a spectrometer using a femtosecond regenerative amplified Ti:sapphire laser apparatus in which the amplifier was seeded with 120 fs laser pulses from an oscillator laser system (with a repetition rate of 1 kHz). Five percent of the amplified 800 nm laser pulses were the source to generate a white beam as the probe pulse, which passed across a sapphire crystal. Afterwards, this laser pulse was separated into two beams before traversing the sample. These two beams passed through the sample and entered the reference spectrometer separately. The corresponding instrument response function (IRF) for fs-TA setup was 300 fs. For the present experiments, 40 mL sample solutions were excited by a 267 nm pump beam in a 2 mm path-length cuvette with an absorbance of 1 at 267 nm [26].

The ns-TA experiments were performed on a LP920 laser flash photolysis spectrophotometer provided by Edinburgh Instruments Ltd (Livingston, British). The probe light source was a 450 W ozone-free Xe arc lamp with 10 Hz to single shot repetition rate, which produced a continuous spectrum between 280 and 800 nm and a flexible sample chamber. The excitation source was a Q-switched Nd:YAG laser system. The entirety of the sample was photoexcited by a 267 nm pump laser pulse, and then a probe beam travelled through the target solution, which was contained in a 1 cm flowing-cell at a specific angle. Next, the transmitted probe light was collected by a detector for further data investigation. Normally, the concentration of the sample solution is determined by a UV absorbance value of 266 nm as 1 [27]. The corresponding IRF for the ns-TA is 5 ns.

### 4.2. Ns-TR^3^ Experiments

The detailed description of ns-TR^3^ measurement could be found in previous reports [23,24,25]. The 266 nm pump laser pulse was formed from the fourth harmonic of a Nd:YAG nanosecond pulsed laser, and the 368.9 nm probe laser pulse was generated from the second Anti-Stokes hydrogen Raman shifted laser line from the second (532 nm) harmonic. A pulse-delay generator controlled these two lasers electronically to change the delay time between them. The time-resolution on this ns-TR^3^ instrument was about 10 ns. After focusing onto the flowing sample solution gently with these two beams, the Raman light was detected through a spectrometer whose grating dispersed the light onto a CCD detector with liquid nitrogen cooling. The known MeCN solvent’s Raman bands (5 cm^−1^ accuracy) were used to calibrate all the obtained Raman spectra, and the target sample solutions were determined by UV absorption value at 266 nm to be 1 in a 1 mm path-length cuvette [28]. The corresponding IRF for the ns-TR^3^ is around 5 ns.

### 4.3. DFT Calculations

DFT calculations performed in this work used the B3LYP method with a 6–311G(d,p) basis set in the PCM solvent model with the selected solvent molecules [29]. A Lorentzian function with a 10 cm^−1^ bandwidth for the vibrational frequencies and a scaling factor of 0.975 were employed to display the predicted normal Raman spectra. The Gaussian 09 program suite [30] was employed, and more details can be found in our previous work [23,24].

## Supplementary Materials

Supplementary materials can be found at https://www.mdpi.com/1422-0067/20/21/5512/s1.
