# Supplementary material for: Time-Resolved Spectroscopic Study of N,N–Di(4–bromo)nitrenium Ions in Acidic Aqueous Solution
Source: Int J Mol Sci. 2019 Nov 5;20(21):5512. doi: 10.3390/ijms20215512 (PMC6862224; doi:10.3390/ijms20215512)
Supplement: Supplementary file 1 [file ijms-20-05512-s001.pdf]

International Journal of *Molecular Sciences*

MDPI

Article

Time-Resolved Spectroscopy Study of the N,N-Di(4-bromo)nitrenium Ions in Acid Solution

Lili Du [1,2,†], Zhiping Yan [2,†], Xueqin Bai [2], Runhui Liang [2] and David Lee Phillips [2,*]

[1] Institute of Life Sciences, Jiangsu University, Zhenjiang 212013, P.R. China; justailleen@gmail.com (L.D.)
[2] Department of Chemistry, The University of Hong Kong, Hong Kong S.A.R., China; mcayzp@gmail.com (Z.Y.); xqbai@hku.hk (X.B.); rhliang5@hku.hk (R.L.)
* Correspondence: phillips@hku.hk (D.L.P.); Tel.: +852-2859-2160 (D.L.P.)
† Those authors contribute equally to this work

Received: date; Accepted: date; Published: date

Supplementary material

Scheme S1. The generation of **DN**……………………………………………………………………………………2

Figure S1. Shown are fs-TA spectra in obtained in 1:1 MeCN: 1 mM $HClO_4$ solution after 267 nm irradiation of **1** (left), and the kinetics at 450 nm and 375 nm (right)…………………………………………………………………2

Table S1. Structural parameter for the intermediate **3**, intermediate **4** and **DN** calculated from the DFT calculations using the B3LYP methods and a 6-311G(d,p) basis set…………………………………………………2

Cartesian coordinates, total energies, and vibrational zero-point energies…………………………………………3

Scheme S1. The generation of **DN**.

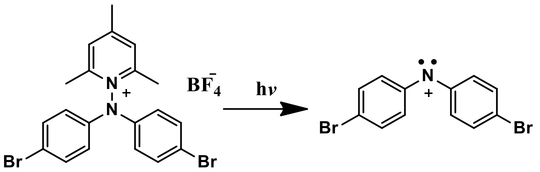

1 **DN**

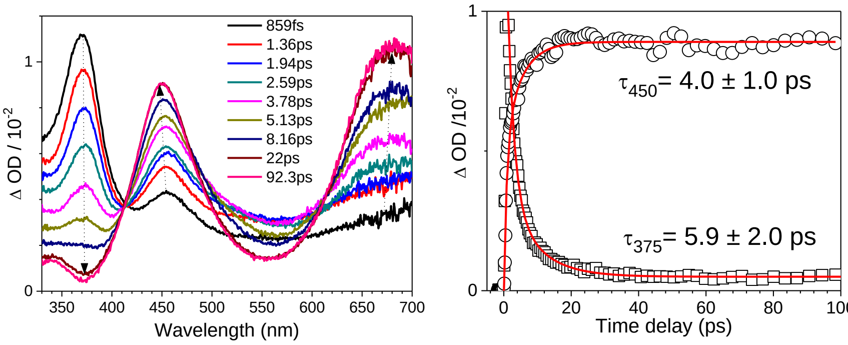

Figure S1. Shown are fs-TA spectra in obtained in 1:1 MeCN: 1 mM $HClO_4$ solution after 267 nm irradiation of **1** (left), and the kinetics at 450 nm and 375 nm (right).

Table S1. Structural parameter for the intermediate **3**, intermediate **4** and **DN** calculated from the DFT calculations using the B3LYP methods and a 6-311G(d,p) basis set.

Bond length (Å)						Bond angles (deg)						Dihedral angles (deg)					
**Intermediate 3**		**Intermediate 4**		**DN**		**Intermediate 3**		**Intermediate 4**		**DN**		**Intermediate 3**		**Intermediate 4**		**DN**	
C1-C2	1.415	C1-C2	1.431	C1-C2	1.431	C1-C2-C3	119.5	C1-C2-C3	119.7	C1-C2-C3	118.6	C1-C2-C3-C4	-2.0	C1-C2-C3-C4	-3.0	C1-C2-C3-C4	-3.4
C2-C3	1.413	C2-C3	1.427	C2-C3	1.433	C2-C3-C4	119.9	C2-C3-C4	119.6	C2-C3-C4	120.2	C2-C3-C4-C5	0.2	C2-C3-C4-C5	-0.1	C2-C3-C4-C5	-0.7
C3-C4	1.380	C3-C4	1.369	C3-C4	1.374	C3-C4-C5	120.0	C3-C4-C5	120.1	C3-C4-C5	119.4	C3-C4-C5-C6	1.3	C3-C4-C5-C6	2.2	C3-C4-C5-C6	2.6
C4-C5	1.403	C4-C5	1.422	C4-C5	1.407	C5-C6-C1	119.5	C5-C6-C1	119.4	C5-C6-C1	118.7	C4-C5-C6-C1	-0.9	C4-C5-C6-C1	-1.1	C4-C5-C6-C1	-0.3
C5-C6	1.404	C5-C6	1.418	C5-C6	1.405	C2-C1-C6	120.5	C2-C1-C6	120.4	C2-C1-C6	120.9	C2-C1-C6-C5	-0.9	C2-C1-C6-C5	-2.0	C2-C1-C6-C5	-3.9
C1-C6	1.378	C1-C6	1.368	C1-C6	1.374	C4-C5-Br15	119.7	C4-C5-Br15	119.5	C4-C5-Br15	118.9	Br15-C5-C6-C1	-179.9	Br15-C5-C6-C1	-179.3	Br15-C5-C6-C1	-178.3
C2-N7	1.382	C2-N7	1.364	C2-N7	1.339	C1-C2-N7	117.5	C1-C2-N7	116.5	C1-C2-N7	115.3	Br15-C5-C4-C3	-179.7	Br15-C5-C4-C3	-179.6	Br15-C5-C4-C3	-179.4
N7-H24	1.015	N7-H24	1.021	C5-Br15	1.883	N7-C8-C13	117.5	N7-C8-C13	116.5	N7-C8-C13	115.3	C1-C2-N7-C8	160.4	C1-C2-N7-C8	164.7	C1-C2-N7-C8	160.8
C5-Br15	1.882	C5-Br15	1.846			C2-N7-C8	131.1	C2-N7-C8	133.8	C2-N7-C8	126.1	C2-N7-C8-C13	160.4	C2-N7-C8-C13	164.7	C2-N7-C8-C13	160.8
						N7-C8-C9	123.0	N7-C8-C9	116.5	N7-C8-C9	126.1	C2-N7-C8-C9	-22.3	C2-N7-C8-C9	-17.7	C2-N7-C8-C9	-22.7
												C3-C2-N7-C8	-22.3	C3-C2-N7-C8	-17.7	C3-C2-N7-C8	-22.7

Cartesian coordinates, total energies, and vibrational zero-point energies for the optimized geometry from the (U)B3LYP/6-311G(d,p) calculations for the compounds and intermediates considered in this paper are given

Radical cation **3**

C	2.34679200	1.78966500	-0.39710600
C	1.25805000	0.98475000	0.01268000
C	1.49913000	-0.33168900	0.46541600
C	2.78474000	-0.83443200	0.46544600
C	3.85060000	-0.03773300	0.02028500
C	3.62828700	1.28199300	-0.40254500
N	-0.00001800	1.55705900	0.00028200
C	-1.25805600	0.98473800	-0.01232200
C	-1.49900000	-0.33186700	-0.46466600
C	-2.78461200	-0.83460200	-0.46487400
C	-3.85060700	-0.03775300	-0.02029200
C	-3.62841500	1.28212700	0.40213900
C	-2.34692000	1.78979300	0.39689500
Br	-5.59816400	-0.73669600	-0.01859100
Br	5.59817000	-0.73666700	0.01832300
H	2.97439300	-1.83484100	0.83117500
H	4.45683800	1.89427600	-0.73270400
H	2.16985900	2.80527300	-0.73437100
H	-2.17009500	2.80552200	0.73384700
H	-4.45706200	1.89452900	0.73183600
H	-2.97416100	-1.83514600	-0.83029300
H	-0.69399200	-0.93164300	-0.86527100
H	0.69421700	-0.93130400	0.86646600
H	-0.00002100	2.57171000	0.00037100

Zero-point correction= 0.177794 (Hartree/Particle)

Sum of electronic and thermal Free Energies= -5665.472200 Hartree

dication **4**

C	-1.49923	-0.33209	-0.46484
C	-1.25805	0.98451	-0.01252
C	-2.34686	1.78967	0.39673
C	-3.62838	1.28213	0.40211
C	-3.85078	-0.03778	-0.02024
C	-2.78489	-0.8347	-0.46492
C	1.2581	0.9844	0.01247
C	1.49917	-0.33217	0.46466
C	2.78489	-0.83476	0.4648

C	3.85075	-0.03775	0.0203
C	3.62838	1.28213	-0.40204
C	2.34682	1.78963	-0.39674
Br	5.59843	-0.73645	0.01846
Br	-5.5984	-0.73647	-0.0184
H	2.97451	-1.83534	0.8301
H	4.45694	1.89467	-0.73169
H	2.16986	2.80535	-0.73365
H	-2.16994	2.80539	0.73367
H	-4.45694	1.89463	0.73183
H	-2.97453	-1.83523	-0.83035
H	-0.69436	-0.93198	-0.86555
H	0.69431	-0.93217	0.86523
N	-0.00006	1.55666	-0.00006
H	0.00005	2.5713	0.00021

Zero-point correction= 0.178577 (Hartree/Particle)

Sum of electronic and thermal Free Energies= -5665.055519 Hartree
